# (4-*tert*-Butyl­phenyl)­acetic acid

**DOI:** 10.1107/S160053680802802X

**Published:** 2008-09-20

**Authors:** Bing-Xin Liu, Yan-Ping Yu, Duan-Jun Xu

**Affiliations:** a Department of Chemistry, Shanghai University, People’s Republic of China; b Department of Chemistry, Zhejiang University, People’s Republic of China

## Abstract

In the title compound, C_12_H_16_O_2_, the plane of the carboxylic acid group is almost perpendicular to the benzene ring [dihedral angle 80.9 (3)°] and the *tert*-butyl unit is disordered over two sets of sites in a 0.503 (6):0.497 (6) ratio. In the crystal structure, centrosymmetric dimers arise from pairs of O—H⋯O hydrogen bonds involving the carboxylic acid groups.

## Related literature

For general background, see: Liu *et al.* (2006 ▶). For a related structure, see: van Koningsveld (1982 ▶).
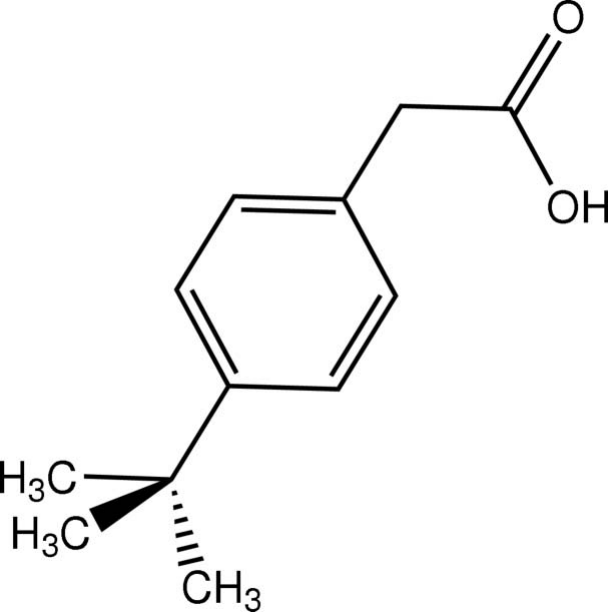


## Experimental

### 

#### Crystal data

C_12_H_16_O_2_
*M* *_r_* = 192.25
Monoclinic, 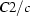

*a* = 11.209 (2) Å
*b* = 12.442 (3) Å
*c* = 17.250 (5) Å
β = 104.625 (12)°
*V* = 2327.8 (10) Å^3^
*Z* = 8
Mo *K*α radiation
μ = 0.07 mm^−1^
*T* = 295 (2) K
0.30 × 0.23 × 0.16 mm

#### Data collection

Bruker APEX CCD diffractometer
Absorption correction: none
5829 measured reflections
2047 independent reflections
1375 reflections with *I* > 2σ(*I*)
*R*_int_ = 0.025

#### Refinement

*R*[*F* ^2^ > 2σ(*F* ^2^)] = 0.074
*wR*(*F* ^2^) = 0.220
*S* = 1.06
2047 reflections
131 parameters
H-atom parameters constrained
Δρ_max_ = 0.30 e Å^−3^
Δρ_min_ = −0.25 e Å^−3^

### 

Data collection: *SMART* (Bruker, 2004 ▶); cell refinement: *SAINT* (Bruker, 2004 ▶); data reduction: *SAINT*; program(s) used to solve structure: *SIR92* (Altomare *et al.*, 1993 ▶); program(s) used to refine structure: *SHELXL97* (Sheldrick, 2008 ▶); molecular graphics: *ORTEP-3* (Farrugia, 1997 ▶); software used to prepare material for publication: *WinGX* (Farrugia, 1999 ▶).

## Supplementary Material

Crystal structure: contains datablocks I, global. DOI: 10.1107/S160053680802802X/hb2766sup1.cif

Structure factors: contains datablocks I. DOI: 10.1107/S160053680802802X/hb2766Isup2.hkl

Additional supplementary materials:  crystallographic information; 3D view; checkCIF report

## Figures and Tables

**Table 1:** Hydrogen-bond geometry (Å, °)

*D*—H⋯*A*	*D*—H	H⋯*A*	*D*⋯*A*	*D*—H⋯*A*
O1—H1⋯O2^i^	0.92	1.74	2.659 (3)	176

Symmetry code: (i) 
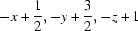
.

**Fig. 1.**
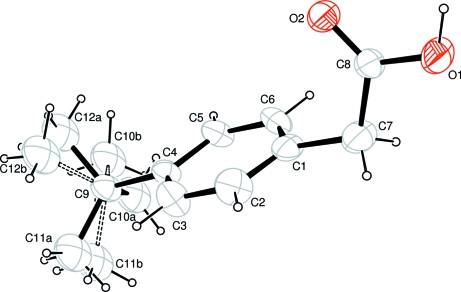
The molecular structure of (I) with 30% probability displacement ellipsoids (arbitrary spheres for H atoms). Double dashed lines indicate one of disordered components.

**Fig. 2.**
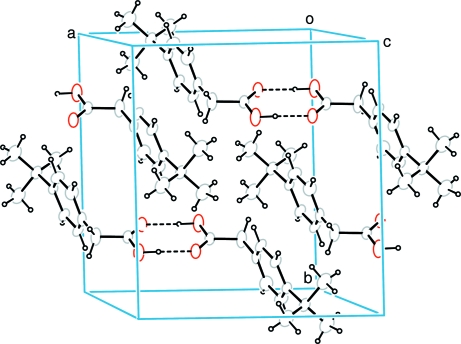
The unit cell packing diagram showing the supra-molecular dimeric structure linked by the hydrogen bonding (dashed lines).

## supplementary crystallographic information

### Comment

As part of investigation on the nature of aromatic stacking (Liu *et al.*, 2006), the title compound, (I), has recently been prepared in Prof. Liu's laboratory by the hydrolization reaction of 2-(4-*tert*-butylphenyl)-1-morpholinoethanethione. Herein we present its X-ray structure (Fig. 1).

The C8—O1 bond distance of 1.309 (3) Å is significantly longer than the C8—O2 bond distance of 1.191 (3) Å. The carboxyl group is nearly perpendicular to the benzene plane, the dihedral angle being 80.9 (3)°. The adjacent molecules are linked together *via* O—H···O hydrogen bonding (Table 1) to form a centrosymmetric supramolecular dimer as shown in Fig. 2, which is comparable to that found in 4-*tert*-butylbenzoic acid (van Koningsveld, 1982).

### Experimental

The title compound was prepared by a hydrolization reaction of 2-(4-*tert*-butylphenyl)-1-morpholinoethanethione (55 g) in a solution containing CH_3_COOH (150 ml), H_2_SO_4_ (25 ml, 98%) and water (30 ml) at 390 K until the reaction mixture changed colour to dark-green. After cooling to room temperature, the solid product was separated from the reaction mixture, and colourless prisms of (I) were obtained by recrystallization of the solid product from an ethanol–water solution (1:1 v/v) after 2 months.

### Refinement

The carboxyl H atom was located in a difference Fourier map and refined as riding in its as-found relative position with *U*_iso_(H) = 1.5*U*_eq_(O). The methyl H atoms were placed in calculated positions with C—H = 0.96 Å and torsion angles were refined to fit the electron density with *U*_iso_(H) = 1.5*U*_eq_(C). The other H atoms were placed in calculated positions with C—H = 0.97 Å (methylene) or 0.93 Å (aromatic), and refined in riding mode with *U*_iso_(H) = 1.2*U*_eq_(C). The tert-butyl group is disordered over two positions. Occupancies were initially refined and converged to 0.503 (6) and 0.497 (6), respectively; in the final cycles of refinement, the occupancies were fixed at 0.5, and displacement parameters for the disordered carbon atoms were constrained to be identical.

### Crystal data

**Table:**

C_12_H_16_O_2_	*F*(000) = 832
*M**_r_* = 192.25	*D*_x_ = 1.097 Mg m^−^^3^
Monoclinic, *C*2/*c*	Mo *K*α radiation, λ = 0.71073 Å
Hall symbol: -C 2yc	Cell parameters from 2180 reflections
*a* = 11.209 (2) Å	θ = 2.8–25.0°
*b* = 12.442 (3) Å	µ = 0.07 mm^−^^1^
*c* = 17.250 (5) Å	*T* = 295 K
β = 104.625 (12)°	Prism, colourless
*V* = 2327.8 (10) Å^3^	0.30 × 0.23 × 0.16 mm
*Z* = 8	

### Data collection

**Table:**

Bruker APEXII CCD diffractometer	1375 reflections with *I* > 2σ(*I*)
Radiation source: fine-focus sealed tube	*R*_int_ = 0.025
graphite	θ_max_ = 25.0°, θ_min_ = 2.4°
Detector resolution: 10 pixels mm^-1^	*h* = −13→12
φ and ω scans	*k* = −11→14
5829 measured reflections	*l* = −20→17
2047 independent reflections	

### Refinement

**Table:**

Refinement on *F*^2^	Primary atom site location: structure-invariant direct methods
Least-squares matrix: full	Secondary atom site location: difference Fourier map
*R*[*F*^2^ > 2σ(*F*^2^)] = 0.075	Hydrogen site location: inferred from neighbouring sites
*wR*(*F*^2^) = 0.220	H-atom parameters constrained
*S* = 1.06	*w* = 1/[σ^2^(*F*_o_^2^) + (0.0943*P*)^2^ + 2.2585*P*] where *P* = (*F*_o_^2^ + 2*F*_c_^2^)/3
2047 reflections	(Δ/σ)_max_ = 0.003
131 parameters	Δρ_max_ = 0.30 e Å^−^^3^
0 restraints	Δρ_min_ = −0.25 e Å^−^^3^

### Special details

**Table:**

Geometry. All e.s.d.'s (except the e.s.d. in the dihedral angle between two l.s. planes) are estimated using the full covariance matrix. The cell e.s.d.'s are taken into account individually in the estimation of e.s.d.'s in distances, angles and torsion angles; correlations between e.s.d.'s in cell parameters are only used when they are defined by crystal symmetry. An approximate (isotropic) treatment of cell e.s.d.'s is used for estimating e.s.d.'s involving l.s. planes.
Refinement. Refinement of *F*^2^ against ALL reflections. The weighted *R*-factor *wR* and goodness of fit *S* are based on *F*^2^, conventional *R*-factors *R* are based on *F*, with *F* set to zero for negative *F*^2^. The threshold expression of *F*^2^ > σ(*F*^2^) is used only for calculating *R*-factors(gt) *etc*. and is not relevant to the choice of reflections for refinement. *R*-factors based on *F*^2^ are statistically about twice as large as those based on *F*, and *R*- factors based on ALL data will be even larger.

### Fractional atomic coordinates and isotropic or equivalent isotropic displacement parameters (Å^2^)

**Table:**

	*x*	*y*	*z*	*U*_iso_*/*U*_eq_	Occ. (<1)
O1	0.3924 (2)	0.7974 (2)	0.56682 (15)	0.1086 (10)	
H1	0.3088	0.7959	0.5607	0.163*	
O2	0.35115 (18)	0.70221 (19)	0.45614 (13)	0.0801 (7)	
C1	0.6042 (2)	0.6780 (3)	0.45859 (19)	0.0733 (9)	
C2	0.6468 (3)	0.5756 (3)	0.4707 (2)	0.0904 (11)	
H2	0.6448	0.5403	0.5179	0.108*	
C3	0.6930 (3)	0.5233 (3)	0.41409 (19)	0.0838 (10)	
H3	0.7216	0.4532	0.4244	0.101*	
C4	0.6984 (2)	0.5702 (2)	0.34325 (15)	0.0582 (7)	
C5	0.6512 (3)	0.6719 (3)	0.33048 (18)	0.0714 (9)	
H5	0.6498	0.7060	0.2823	0.086*	
C6	0.6056 (3)	0.7253 (3)	0.3870 (2)	0.0819 (10)	
H6	0.5753	0.7948	0.3764	0.098*	
C7	0.5608 (3)	0.7390 (4)	0.5219 (2)	0.1051 (14)	
H7A	0.5971	0.7062	0.5735	0.126*	
H7B	0.5917	0.8120	0.5237	0.126*	
C8	0.4244 (3)	0.7432 (3)	0.51024 (17)	0.0653 (8)	
C9	0.7547 (3)	0.5128 (3)	0.28348 (18)	0.0732 (9)	
C10A	0.8271 (11)	0.6028 (9)	0.2430 (6)	0.1125 (15)	0.497 (6)
H10A	0.8575	0.5698	0.2015	0.169*	0.497 (6)
H10B	0.8950	0.6315	0.2833	0.169*	0.497 (6)
H10C	0.7713	0.6598	0.2207	0.169*	0.497 (6)
C11A	0.8538 (13)	0.4343 (11)	0.3166 (9)	0.1125 (15)	0.497 (6)
H11A	0.8187	0.3714	0.3344	0.169*	0.497 (6)
H11B	0.9124	0.4660	0.3611	0.169*	0.497 (6)
H11C	0.8944	0.4147	0.2759	0.169*	0.497 (6)
C12A	0.6605 (9)	0.4711 (9)	0.2153 (6)	0.1125 (15)	0.497 (6)
H12A	0.6206	0.5299	0.1829	0.169*	0.497 (6)
H12B	0.6006	0.4309	0.2344	0.169*	0.497 (6)
H12C	0.6985	0.4251	0.1838	0.169*	0.497 (6)
C10B	0.7561 (11)	0.5733 (9)	0.2101 (6)	0.1125 (15)	0.503 (6)
H10D	0.7923	0.5297	0.1761	0.169*	0.503 (6)
H10E	0.8037	0.6377	0.2243	0.169*	0.503 (6)
H10F	0.6732	0.5917	0.1823	0.169*	0.503 (6)
C11B	0.8802 (13)	0.4692 (11)	0.3269 (8)	0.1125 (15)	0.503 (6)
H11D	0.8717	0.4232	0.3698	0.169*	0.503 (6)
H11E	0.9342	0.5279	0.3482	0.169*	0.503 (6)
H11F	0.9142	0.4289	0.2900	0.169*	0.503 (6)
C12B	0.6679 (9)	0.4091 (9)	0.2531 (6)	0.1125 (15)	0.503 (6)
H12D	0.5836	0.4318	0.2348	0.169*	0.503 (6)
H12E	0.6756	0.3592	0.2965	0.169*	0.503 (6)
H12F	0.6931	0.3750	0.2099	0.169*	0.503 (6)

### Atomic displacement parameters (Å^2^)

**Table:**

	*U*^11^	*U*^22^	*U*^33^	*U*^12^	*U*^13^	*U*^23^
O1	0.0599 (14)	0.172 (3)	0.0991 (17)	−0.0057 (14)	0.0303 (12)	−0.0708 (17)
O2	0.0532 (12)	0.1151 (18)	0.0758 (14)	−0.0020 (11)	0.0231 (10)	−0.0310 (12)
C1	0.0408 (15)	0.112 (3)	0.0682 (19)	0.0018 (15)	0.0160 (13)	−0.0220 (18)
C2	0.098 (3)	0.114 (3)	0.068 (2)	0.007 (2)	0.0381 (19)	0.0096 (19)
C3	0.105 (3)	0.078 (2)	0.078 (2)	0.0180 (19)	0.0417 (19)	0.0145 (17)
C4	0.0506 (15)	0.0689 (17)	0.0574 (15)	0.0100 (13)	0.0178 (12)	0.0069 (13)
C5	0.0659 (18)	0.084 (2)	0.0675 (18)	0.0235 (16)	0.0221 (14)	0.0174 (15)
C6	0.064 (2)	0.086 (2)	0.094 (2)	0.0292 (16)	0.0180 (17)	−0.0029 (18)
C7	0.0542 (19)	0.171 (4)	0.091 (2)	0.003 (2)	0.0214 (17)	−0.053 (3)
C8	0.0546 (17)	0.086 (2)	0.0594 (16)	0.0042 (14)	0.0218 (14)	−0.0110 (15)
C9	0.0687 (19)	0.091 (2)	0.0630 (17)	0.0215 (16)	0.0231 (15)	0.0022 (16)
C10A	0.116 (3)	0.136 (4)	0.098 (3)	0.029 (3)	0.050 (3)	−0.008 (2)
C11A	0.116 (3)	0.136 (4)	0.098 (3)	0.029 (3)	0.050 (3)	−0.008 (2)
C12A	0.116 (3)	0.136 (4)	0.098 (3)	0.029 (3)	0.050 (3)	−0.008 (2)
C10B	0.116 (3)	0.136 (4)	0.098 (3)	0.029 (3)	0.050 (3)	−0.008 (2)
C11B	0.116 (3)	0.136 (4)	0.098 (3)	0.029 (3)	0.050 (3)	−0.008 (2)
C12B	0.116 (3)	0.136 (4)	0.098 (3)	0.029 (3)	0.050 (3)	−0.008 (2)

### Geometric parameters (Å, °)

**Table:**

O1—C8	1.309 (3)	C9—C11B	1.517 (15)
O1—H1	0.9169	C9—C12B	1.621 (11)
O2—C8	1.191 (3)	C9—C10A	1.639 (11)
C1—C2	1.358 (5)	C10A—H10A	0.9600
C1—C6	1.371 (5)	C10A—H10B	0.9600
C1—C7	1.508 (4)	C10A—H10C	0.9600
C2—C3	1.379 (4)	C11A—H11A	0.9600
C2—H2	0.9300	C11A—H11B	0.9600
C3—C4	1.369 (4)	C11A—H11C	0.9600
C3—H3	0.9300	C12A—H12A	0.9600
C4—C5	1.368 (4)	C12A—H12B	0.9600
C4—C9	1.517 (4)	C12A—H12C	0.9600
C5—C6	1.381 (4)	C10B—H10D	0.9600
C5—H5	0.9300	C10B—H10E	0.9600
C6—H6	0.9300	C10B—H10F	0.9600
C7—C8	1.491 (4)	C11B—H11D	0.9600
C7—H7A	0.9700	C11B—H11E	0.9600
C7—H7B	0.9700	C11B—H11F	0.9600
C9—C12A	1.463 (10)	C12B—H12D	0.9600
C9—C10B	1.476 (10)	C12B—H12E	0.9600
C9—C11A	1.480 (15)	C12B—H12F	0.9600
			
C8—O1—H1	111.6	C10B—C9—C12B	105.3 (5)
C2—C1—C6	117.3 (3)	C4—C9—C12B	106.0 (4)
C2—C1—C7	121.7 (3)	C11B—C9—C12B	106.3 (6)
C6—C1—C7	121.0 (4)	C12A—C9—C10A	103.5 (6)
C1—C2—C3	120.9 (3)	C11A—C9—C10A	102.2 (7)
C1—C2—H2	119.5	C4—C9—C10A	107.7 (4)
C3—C2—H2	119.5	C9—C10A—H10A	109.5
C4—C3—C2	122.7 (3)	C9—C10A—H10B	109.5
C4—C3—H3	118.7	C9—C10A—H10C	109.5
C2—C3—H3	118.7	C9—C11A—H11A	109.5
C5—C4—C3	115.8 (3)	C9—C11A—H11B	109.5
C5—C4—C9	122.4 (3)	C9—C11A—H11C	109.5
C3—C4—C9	121.7 (3)	C9—C12A—H12A	109.5
C4—C5—C6	121.9 (3)	C9—C12A—H12B	109.5
C4—C5—H5	119.0	C9—C12A—H12C	109.5
C6—C5—H5	119.0	C9—C10B—H10D	109.5
C1—C6—C5	121.3 (3)	C9—C10B—H10E	109.5
C1—C6—H6	119.4	H10D—C10B—H10E	109.5
C5—C6—H6	119.4	C9—C10B—H10F	109.5
C8—C7—C1	115.3 (3)	H10D—C10B—H10F	109.5
C8—C7—H7A	108.5	H10E—C10B—H10F	109.5
C1—C7—H7A	108.5	C9—C11B—H11D	109.5
C8—C7—H7B	108.5	C9—C11B—H11E	109.5
C1—C7—H7B	108.5	H11D—C11B—H11E	109.5
H7A—C7—H7B	107.5	C9—C11B—H11F	109.5
O2—C8—O1	122.7 (3)	H11D—C11B—H11F	109.5
O2—C8—C7	124.9 (3)	H11E—C11B—H11F	109.5
O1—C8—C7	112.4 (3)	C9—C12B—H12D	109.5
C12A—C9—C11A	113.3 (6)	C9—C12B—H12E	109.5
C12A—C9—C4	112.0 (4)	H12D—C12B—H12E	109.5
C10B—C9—C4	116.0 (4)	C9—C12B—H12F	109.5
C11A—C9—C4	116.6 (6)	H12D—C12B—H12F	109.5
C10B—C9—C11B	113.3 (7)	H12E—C12B—H12F	109.5
C4—C9—C11B	109.0 (5)		

### Hydrogen-bond geometry (Å, °)

**Table:**

*D*—H···*A*	*D*—H	H···*A*	*D*···*A*	*D*—H···*A*
O1—H1···O2^i^	0.92	1.74	2.659 (3)	176

Symmetry codes: (i) −*x*+1/2, −*y*+3/2, −*z*+1.
